# A hitchhiker's guide to single‐cell epigenomics: Methods and applications for cancer research

**DOI:** 10.1002/ijc.35307

**Published:** 2024-12-22

**Authors:** Marta Moreno‐Gonzalez, Isabel Sierra, Jop Kind

**Affiliations:** ^1^ Hubrecht Institute, Royal Netherlands Academy of Arts and Sciences (KNAW) & University Medical Center Utrecht Utrecht The Netherlands; ^2^ Oncode Institute Utrecht The Netherlands; ^3^ Department of Molecular Biology Radboud Institute for Molecular Life Sciences, Radboud University Nijmegen The Netherlands

**Keywords:** cancer, chromatin, histone modifications, single‐cell epigenomics, tumor heterogeneity

## Abstract

Genetic mutations are well known to influence tumorigenesis, tumor progression, treatment response and relapse, but the role of epigenetic variation in cancer progression is still largely unexplored. The lack of epigenetic understanding in cancer evolution is in part due to the limited availability of methods to examine such a heterogeneous disease. However, in the last decade the development of several single‐cell methods to profile diverse chromatin features (chromatin accessibility, histone modifications, DNA methylation, etc.) has propelled the study of cancer epigenomics. In this review, we detail the current landscape of single‐omic and multi‐omic single‐cell methods with a particular focus on the examination of histone modifications. Furthermore, we provide recommendations on both the application of these methods to cancer research and how to perform initial computational analyses. Together, this review serves as a referential framework for incorporating single‐cell methods as an important tool for tumor biology.

## Graphical abstract

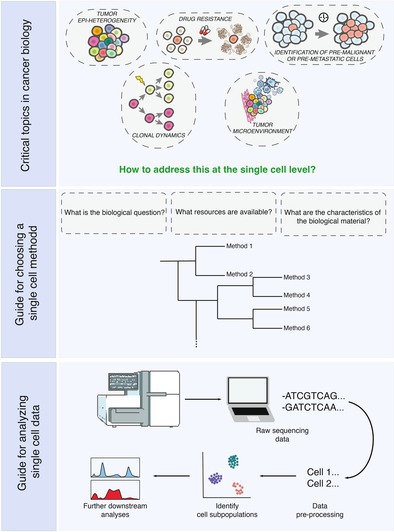

## 1 INTRODUCTION

Studying the regulatory mechanisms controlling gene expression is key to understanding the onset and progression of diseases like cancer.^1^ Unlike genetic alterations, epigenetic changes control gene expression through chemical modifications that change chromatin compaction without changing the underlying DNA sequence. These changes include DNA methylation,^2^ histone post‐translational modifications (hPTMs),^3^ nucleosome localization, and 3D chromatin organization. Alterations in chromatin compaction affect accessibility for transcription machinery and consequently gene expression.

The role of genetic alterations in cancer development has been widely studied,^4^ but the roles of epigenetic mechanisms in tumorigenesis, progression, and metastasis remain less understood.^1^ Mutations in components of chromatin remodeling complexes are common across cancers, with over 25% of cancers presenting mutations in components of the SWI‐SNF complexes.^5^ A recent study in *Drosophila* suggests that epigenetic dysregulation alone can drive tumorigenic cell fate.^6^ Changes in DNA methylation patterns are most prevalently studied for their role in cancer development,^7^, ^8^, ^9^ however, aberrant hPTMs patterns also play a role. For example, H3K4 methylation levels vary during tumor progression in renal cancers,^10^ and H3K27me3, which is being explored as a potential therapeutic target,^11^ was identified as one of the main epigenetic drivers of cancer.^12^

**FIGURE 1:**
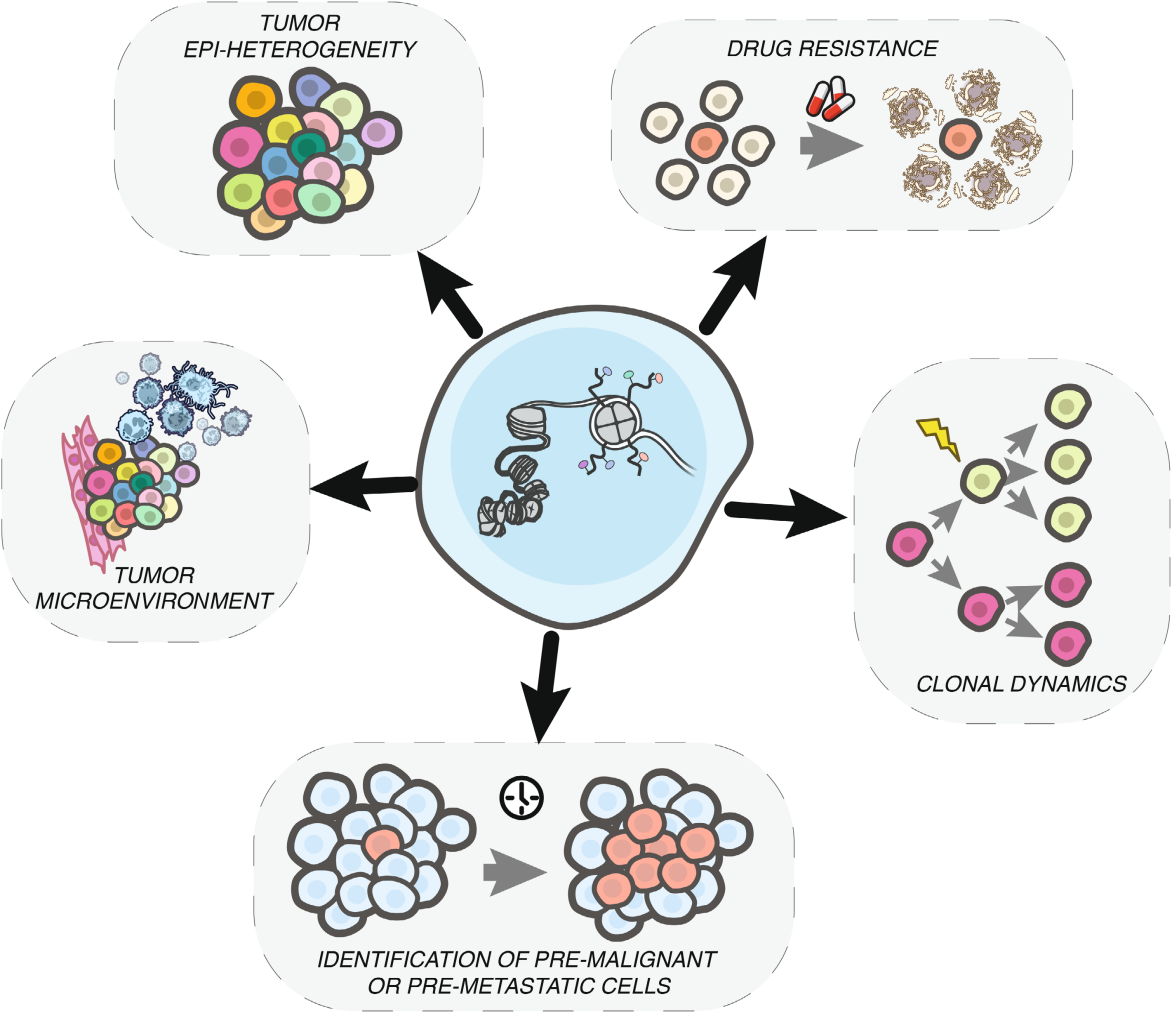
Potential uses of single‐cell epigenomics in cancer research. Single‐cell epigenomic methods can interrogate the chromatin state (center—histone tail modifications, accessibility) of an individual cell. This high resolution may be key to understanding several aspects of tumorigenesis and metastasis, such as tumor epi‐heterogeneity, the influence of tumor microenvironment on epigenetic adaptation, epigenetic characteristics of pre‐malignant or pre‐metastatic cells, the effect of chromatin variants on clonal dynamics, and the epigenetic aspects of drug resistance.

**FIGURE 2:**
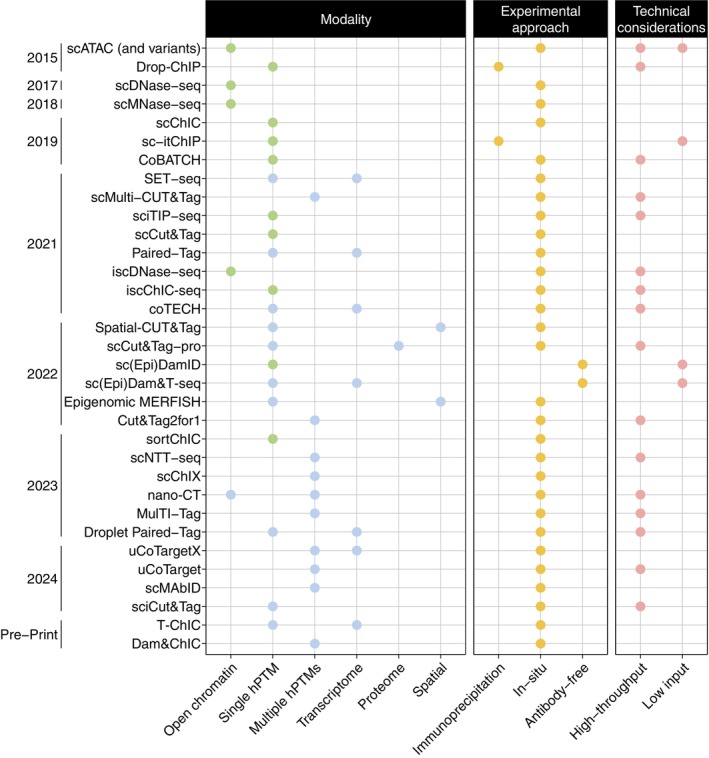
Overview of single‐cell methods for chromatin profiling. Overview of different methods to profile chromatin features at a single‐cell level, ordered by year of publication. Methods are distinguished by their ability to profile either a single modality (Modality column, green dots) or multiple modalities (Modality column, blue dots). Information regarding the underlying methodology of each approach (Experimental approach column, yellow dots) and if the technique is appropriate for high or low throughput (Technical considerations column, red dots) is also included.

While we have some insights into the epigenetic landscape of tumors at the bulk level, the lack of techniques to profile hPTMs at the single‐cell level has left us with a limited understanding of intra‐tumor epigenetic heterogeneity. Several studies have examined chromatin accessibility in single cancer cells, but a more systemic analysis of epigenetic regulation is still needed.^13^, ^14^ Epigenomic single‐cell studies could provide key insights into the role of epigenetic mechanisms on cell heterogeneity^15^ and tumor evolution,^16^ among others (Figure 1). In the last couple of years, we have seen a drastic increase in the number of methods to profile hPTMs at a single‐cell level, both in a single‐omic and multi‐omic context. In this review, we aim to expand on recent discussions^17^, ^18^ of single‐cell epigenomics to include these new methods (summarized in Figure 2) and provide recommendations for application to cancer‐centered research questions.

## 2 PROFILING CHROMATIN FEATURES AT A SINGLE‐CELL LEVEL

### 2.1 Chromatin accessibility

Chromatin accessibility is the result of the interplay of various epigenetic mechanisms that collectively alter the compaction of the DNA in the nucleus. Profiling chromatin accessibility can be used to identify active and inactive regions that play a role in development and disease, as well as transcription factors that might regulate these processes.^19^ This profiling is possible due to chromatin having differential accessibility to various enzymes. As each enzyme has particular biases, care should be taken when selecting the approach and interpreting the resulting data. Here we describe the most commonly used approaches to profile chromatin accessibility at a single‐cell level. We refer to previous reviews^20^, ^21^, ^22^ for more detailed discussions on this topic.

Several methods exist to profile chromatin accessibility at a single‐cell level. These technologies are built on long‐standing assays using the enzymes DNase I and MNase to digest chromatin.^23^, ^24^, ^25^ DNase I is an endonuclease that preferentially introduces double‐stranded breaks in open chromatin to provide a read‐out of accessibility, while MNase cleaves and digests regions not protected by nucleosomes leaving only nucleosome‐protected regions for sequencing. Both assays were adapted to the single‐cell level in the form of scDNase‐seq,^26^ iscDNase‐seq,^27^ and scMNase‐seq.^28^ However, limitations exist due to the nature of the enzymes used. Both DNase I and MNase‐based protocols are sensitive to enzyme concentration, and require titration for each cell type. DNase I shows a sequence bias with preference for sites near CpG methylation islands, while MNase preferentially cleaves A/T‐rich DNA sequences.^22^

Currently, the most popular assay to map chromatin accessibility is ATAC‐seq, which takes advantage of the hyperactive DNA transposase Tn5 to insert sequencing adapters in open chromatin regions.^29^ A wide range of scATAC‐seq‐based methods were developed in the last decades with different levels of starting sample requirements, throughput capacity, coverage per cell, cost per cell, etc. A recent effort by De Rop et al. provided a comprehensive and systematic benchmark of scATAC‐seq‐based protocols.^30^ A major advantage of ATAC‐seq‐based methods is that they tend to be simpler protocols than other open chromatin profiling methods^22^ and commercial platforms are available to facilitate ease‐of‐use for novices in the field.

### 2.2 Histone modifications

hPTMS can influence chromatin accessibility and gene expression through multiple mechanisms and must be carefully regulated.^31^ Mutations in genes regulating hPTMs are widely associated with cancer, and therefore profiling hPTMs may provide important insights into how epigenetic processes affect cancer progression and metastasis.^6^, ^32^ Methods to profile hPTMs at single‐cell levels can be grouped into three main approaches: immunoprecipitation‐based, in‐situ (through either Tn5 or MNase; antibody‐dependent), and antibody‐free (Figure 2).

Immunoprecipitation‐based methods combine conventional chromatin immunoprecipitation (ChIP) methods with single‐cell tagging strategies. In ChIP, antibodies specifically bind to the hPTM of interest, and a subsequent antibody pulldown enriches for DNA associated with the target modification. The success of profiling hPTM with these methods is therefore reliant on antibody quality, as the use of inferior‐quality antibodies results in nonspecific binding and increasing background noise in the resulting dataset. Recent examples include Drop‐ChIP,^33^ a high throughput method that uses droplet barcoding to tag single cells. However, the coverage per cell is limited (~1000 reads/cell). An alternative with higher coverage per cell is sc‐itChIP^34^ (~9000 reads/cell), yet low throughput limits the use of this method for most research questions in cancer research.

More recently, several antibody‐dependent methods take advantage of the ability of the Tn5 transposase to cleave and insert adapter sequences into DNA^35^ to profile hPTMs at the single‐cell level. Tn5 can be fused to protein A (pA), which interacts with secondary antibodies, allowing for the insertion of adapters at antibody binding sites. It should be noted that a limitation of methods reliant on Tn5 is that they can introduce biases via non‐specific insertion to open chromatin. CUT&Tag,^36^ which uses this proteinA‐Tn5 (pA‐Tn5) fusion protein approach, was directly adapted for single‐cell profiling in the form of scCUT&Tag.^37^ However, the original paper reported limited coverage per cell (roughly 600 unique fragments/cell). sciCUT&Tag^38^ combines the scCUT&Tag protocol with combinatorial barcoding for higher throughput while doubling the number of reads per cell. Similarly, CoBATCH^39^ uses combinatorial barcoding with a shortened one‐step library preparation for single‐cell tagging, allowing for high throughput with a higher coverage per cell (~12,000 reads/cell). sciTIP‐seq^40^ combines the use of pA‐Tn5 with linear amplification via T7 RNA polymerase to largely increase the number of unique reads per cell (see Table 1). Together with combinatorial barcoding, this approach provides a high‐throughput, low‐cost alternative. Additionally, ChIL‐seq^65^ is a low‐throughput bulk approach that could be used for single cells, and it might be particularly attractive for profiling hPTMs in samples with limited material, such as rare metastasis spots. While it has shown potential for single‐cell adaptation, this methodology would require further optimization to increase throughput and become more cost‐effective. Also, the elaborate protocol takes 3–4 days to complete and includes several buffer exchange steps that can lead to some cellular loss.

**TABLE 1:** Technical aspects of single‐cell methods.

Modalities	Technique	Single‐cell tech used	Length of protocol	Specialized reagents	Specialized equipment	polyA or full‐length	Maximum reported (hPTM/accessibility) reads per cell	Citation
Chromatin accessibility	iscDNase‐seq	Combinatorial	Short	DNase		NA	**	Gao et al.^27^
Chromatin accessibility	scDNase‐seq	Plate‐based^a^	Short	DNase	FACS	NA	*****	Cooper et al.^26^
Chromatin accessibility	scMNase‐seq	Plate‐based^a^	Short	MNase	FACS	NA	****	Gao et al.^41^
Multiple hPTM	Cut&Tag2for1	Combinatorial	Short	pAG‐Tn5	nanowell dispensing robot	NA	Not reported	Janssens et al.^42^
Multiple hPTM	Dam&ChIC	Plate‐based	Long	transgenic insertion of Dam construct; pA‐Mnase	FACS, nanoliter dispensing robots	NA	***	Kefalopoulou et al.^43^
Multiple hPTM	MulTI‐Tag	Combinatorial	Short	pA‐Tn5; conjugated antibodies	nanowell dispensing robot	NA	*	Meers et al.^44^
Multiple hPTM	nano‐CT	Droplet	Medium	nanobody‐Tn5	10X chromium	NA	***	Bartosovic et al.^46^
Multiple hPTM	scChIX	Plate‐based	Long	pA‐MNase	FACS, nanoliter dispensing robots	NA	**	Yeung et al.^45^
Multiple hPTM	scMAbID	Plate‐based	Long	conjugated antibodies	FACS, nanoliter dispensing robots	NA	*	Lochs et al.^47^
Multiple hPTM	scMulti‐CUT&Tag	Droplet	Short	barcoded pA‐Tn5 + antibody	10X chromium	NA	*	Gopalan et al.^48^
Multiple hPTM	scNTT‐seq	Droplet	Medium	nanobody‐Tn5	10X chromium	NA	**	Stuart et al.^49^
Multiple hPTM	uCoTarget	Combinatorial	Short	pA‐Tn5		NA	**	Xiong et al.^50^
Multiple hPTM + RNA	uCoTargetX	Combinatorial	Short	pA‐Tn5		polyA	**	Xiong et al.^50^
Single hPTM	CoBATCH	Combinatorial	Short	pA‐Tn5		NA	***	Wang et al.^39^
Single hPTM	Drop‐ChIP	Droplet	Medium	MNase	microfluidics device	NA	**	Rotem et al.^33^
Single hPTM	iscChIC‐seq	Combinatorial	Medium	pA‐MNase	FACS	NA	****	Ku et al.^51^
Single hPTM	sc(Epi)DamID	Plate‐based	Long	transgenic insertion of Dam‐POI	FACS, nanoliter dispensing robots	NA	***	Rang et al.^52^ (scEpiDamID); Kind et al.^53^ (scDamID)
Single hPTM	sc‐itChIP	Combinatorial	Short	Tn5 transposase		NA	***	Ai et al.^34^
Single hPTM	scChIC	Plate‐based^a^	Short	pA‐MNase	FACS	NA	***	Ku et al.^54^
Single hPTM	scCut&Tag	Droplet	Short	pA‐Tn5	10X chromium	NA	*	Bartosovic et al.^37^
Single hPTM	sciCut&Tag	Combinatorial	Short	pA‐Tn5	nanowell dispensing robot	NA	**	Janssens et al.^38^
Single hPTM	sciTIP‐seq	Combinatorial	Short	ME‐T7 (pA‐Tn5 with T7 promoter)		NA	****	Bartlett et al.^40^
Single hPTM	sortChIC	Plate‐based	Long	pA‐MNase	FACS, nanoliter dispensing robots	NA	****	Zeller et al.^55^
Single hPTM + Proteins	scCut&Tag‐pro	Droplet	Short	conjugated antibodies; pAG‐Tn5	10X chromium	NA	*	Zhang et al.^56^
Single hPTM + RNA	coTECH	Combinatorial	Medium	pA‐Tn5		polyA	**	Xiong et al.^57^
Single hPTM + RNA	Droplet Paired‐Tag	Droplet	Short	pA‐Tn5	10X chromium	polyA	**	Xie et al.^58^
Single hPTM + RNA	Paired‐Tag	Combinatorial	Medium	pA‐Tn5		polyA	**	Zhu et al.^59^
Single hPTM + RNA	sc(Epi)Dam&T‐seq	Plate‐based	Long	transgenic insertion of Dam‐POI	FACS, nanoliter dispensing robots	polyA	***	Rang et al.^52^ (scEpiDam&T); Rooijers et al.^60^ (scDam&T‐seq)
Single hPTM + RNA	SET‐seq	Plate‐based	Short	pA‐Tn5		polyA	***	Sun et al.^61^
Single hPTM + RNA	T‐ChIC	Plate‐based	Long	pA‐Mnase	FACS, nanoliter dispensing robots	full‐length	****	Zeller et al.^62^
Spatial hPTM	Epigenomic MERFISH	Combinatorial	Medium	pA‐Tn5	microscope	NA	NA	Lu et al.^63^
Spatial hPTM	Spatial‐CUT&Tag	Combinatorial	Short	pA‐Tn5	microfluidics device; microscope	NA	NA	Deng et al.^64^

*Note*: The table displays six metrics for each technique discussed: (1) single‐cell technology each method is based on; (2) relative length of each protocol; (3) specialized reagents required; (4) specialized equipment required; (5) for methods that include a transcriptomic output, capture of mRNA or total RNA; (6) maximum reported reads per single‐cell (*≤999; **1000–6999; ***7000–30,000; ****30,000–99,999; *****>100,000).

^a^ Manual well‐based methods.

Some other antibody‐dependent methods use a pA‐MNase fusion protein instead of pA‐Tn5, which when bound to a target antibody will cut the surrounding DNA sequences leaving the fragments of interest for subsequent library preparation. In bulk, this pA‐MNase approach was first used in ChIC^66^ to profile transcription factor binding, and in CUT&RUN^67^, ^68^ to profile both transcription factor binding and hPTMs. scChIC‐seq^54^ is a single‐cell approach that measures hPTMs through either a pA‐MNase fusion or by directly conjugating antibodies with MNase, eliminating the need for protein A. A higher throughput version is iscChIC‐seq,^51^ which uses combinatorial barcoding for single‐cell tagging. Another pA‐MNase‐based method is sortChIC,^55^ which shows higher specificity than scChIC‐seq and has the option to enrich for rare cell subpopulations via surface staining. While it is low‐throughput due to being plate‐based, this strategy might be particularly interesting for the study of cell subpopulations with known surface markers, such as specific immune cell types.

In contrast to the above, DamID‐based approaches rely on the fusion of a protein of interest (POI) with the *E. coli* deoxyadenosine methylase (Dam) instead of antibodies.^69^ Subsequently, Dam methylates adenine residues in GATC sequences near the binding site of the POI. While Dam cannot be directly fused to post‐translationally modified proteins, it can be fused to chromatin‐binding modules, which allow the profiling of hPTMs. This form of DamID, termed EpiDamID,^52^ can be used with other DamID‐based approaches like scDamID‐seq,^53^ a plate‐based single‐cell epigenomic profiling method. (Epi)DamID‐based approaches can have genomic biases for open chromatin (which can be normalized against the readout of an untethered Dam protein^52^) and GATC‐rich sequences, and require cells to be genetically engineered to express a DamID construct. Despite this, DamID‐based methods are particularly suited for low input scenarios due to minimal sample handling and avoid reliance on antibody quality. A clear limitation of this technology is the requirement for genetic engineering which restricts this method to cellular systems or animal models.

## 3 SINGLE‐CELL HPTM PROFILING IN A MULTI‐OMIC CONTEXT

Most of the previously described methods are designed to profile a single hPTM per cell. However, in the last few years, the field has evolved towards multi‐omic approaches that allow the simultaneous study of several epigenetic layers (Figure 2). While powerful, researchers should take note that increasing the number of targets usually comes at a cost of increased data sparsity (see Table 1—max reported reads per cell). This will be discussed again later in the review. In this section, we will describe the currently existing multi‐omic methods targeting hPTMs.

### 3.1 Profiling multiple hPTMs within the same cell

Different chromatin states arise as a result of the combination and interaction of epigenetic factors like hPTMs at individual loci. To study multi‐factorial chromatin landscapes, several methods have been developed to allow for the joint profiling of several hPTMs in single cells.

Both scNTT‐seq^49^ and nano‐CT^46^ use a pA‐Tn5 fused to nanobodies that confer antibody specificity to specific secondary antibodies. nanoCT has a higher coverage per cell and also allows the profiling of open chromatin. In scMulti‐CUT&Tag,^48^ MulTI‐Tag^44^ and uCoTarget,^50^ antibodies are complexed to pA‐Tn5 which allows for antibody‐specific demultiplexing due to the direct ligation of barcodes to DNA fragments in cells. scMAbID^47^ also uses barcoded antibodies, however, it relies on proximity ligation instead of Tn5‐based tagmentation. It should be noted that all these methods have a low coverage per hPTM per cell, with only nanoCT and uCoTarget containing more than a couple thousand reads per hPTM (Table 1). Furthermore, NTT‐seq and nanoCT allow the co‐profiling of up to three hPTMs at a time, while both scMAbID and uCoTarget allow for higher complexity. Finally, Dam&ChIC^43^ combines sc(Epi)DamID with sortChIC to jointly profile two hPTMs with high coverage per cell per modality (~10,000 reads/cell).

In addition to these experimental developments, recent computational advances allow for deconvolution of jointly profiled hPTMs at the single‐cell level. scChIX^45^ can be used to deconvolute sortChIC datasets incubated with two antibodies through the probabilistic assignment of individual fragments. However, it requires training single‐incubation datasets for each target antibody, which might make it unsuitable with rare samples where input is limited or there are high levels of heterogeneity. In contrast, CUT&Tag2for1^42^ deconvolutes active and repressive chromatin regions from CUTAC (a modified version of CUT&Tag) datasets using different tagmentation densities and fragment sizes without training data. Nonetheless, this method is based on the clear differences between RNA polymerase II and H3K27me3 CUTAC profiles, and it might not be applicable when the targeted chromatin features do not exhibit such clear differences.

### 3.2 Joint hPTMs and transcriptomics

Chromatin modifications influence cellular identity through cell‐type‐specific gene expression. Methods like 10X multiome allow the study of both chromatin accessibility and gene expression within the same cell.^70^ Here, we will focus on approaches that allow for profiling of transcriptomics with hPTMs, enabling a better understanding of how hPTMs regulate gene expression. Furthermore, this transcriptional information can then be used to in silico classify cells into various clusters according to their transcriptional identity.

Several methods have adapted scCUT&Tag for joint profiling of hPTMs and gene expression. Paired‐Tag^59^ expands on the previously published Paired‐seq^71^ protocol, a joint open chromatin and transcriptomic readout, to simultaneously profile hPTMs and nuclear RNA. Subsequently, droplet Paired‐Tag,^58^ uses droplet barcoding to allow for higher throughput and achieves a higher coverage per cell (~5–10 times more reads per modality in mouse embryonic stem cells). Another adaptation of CUT&Tag is SET‐seq,^61^ which specifically allows for the joint profiling of hPTMs and cytoplasmic RNA. Despite the lower throughput, SET‐seq is suitable for scenarios where sorting or selecting cells via a high‐throughput approach is not possible.

Apart from CUT&Tag, other single‐omic approaches were modified to include transcriptomic readouts: sc(Epi)Dam&T‐seq^52^, ^60^ combines (Epi)DamID with CELseq2,^72^ and CoTECH^57^ combines COBATCH with an RNA readout. While sc(Epi)Dam&T‐seq is only available as a plate‐based approach, the CoTECH protocol can be used in a combinatorial indexing form or as a droplet‐based method^62^ for decreased manual processing time. Another recent addition to this toolbox is T‐ChIC, which combines sortChIC and VASA‐seq^73^ to jointly profile hPTMs and full‐length transcripts, yet this method is plate‐based and has limited throughput.

Notably, the methods described above only allow for the profiling of one hPTM with gene expression. As these techniques profile a single hPTM, it is not possible to study how hPTM co‐occupancy and interactions affect gene expression. This is achieved by the recently developed uCoTargetX,^50^ which merges uCoTarget and Smart‐seq2^74^ to provide a joint readout of multiple hPTMs and polyadenylated RNA. However, data sparsity is still a concern and should be considered when choosing this technique with the specific research question in mind.

### 3.3 Joint hPTMs and proteomics

While joint chromatin features and RNA profiling can provide information on the regulatory role of hPTMs, examination of proteins is also useful. scCUT&Tag‐pro^56^ combines CUT&Tag‐derived hPTM readouts with antibody‐mediated surface protein identity as shown in CITE‐seq,^75^ providing an alternative way to study the functional role of hPTMs. This method can be combined with both the 10X chromium technology and antibody‐mediated cell hashing^76^ to achieve extremely high throughput.

### 3.4 Combining hPTMs with spatial information

Finally, adding spatial information to hPTM readouts can provide additional information on how chromatin states affect cell‐type specification and cell states in complex tissues. Spatial‐CUT&Tag^64^ combines scCUT&Tag with in‐tissue deterministic barcoding to obtain single‐cell spatial hPTM information from tissue samples. Spatial CUT&Tag was further developed to also include transcriptomic readout in the form of spatial CUT&Tag‐RNA.^77^ While this technology does not yet provide single‐cell resolution, future developments might allow us to jointly obtain spatial, epigenome, and transcriptome information at a single‐cell level. Epigenomic MERFISH^63^ is a targeted approach that uses Tn5‐based tagmentation to capture epigenetic marks of interest, amplifies the tagged DNA via in situ transcription, and then detects the resulting mRNAs via MERFISH imaging. While it allows for a much higher resolution than scCUT&Tag, allowing even sub‐nuclear analysis, it requires prior knowledge for selecting epigenomic loci, making it unable to unbiasedly profile the epigenome genome‐wide.

## 4 CHOICE OF TECHNIQUE BASED ON SAMPLE CONSIDERATIONS

As demonstrated above, a significant number of techniques exist to examine biological data at the single‐cell level. Thus, choosing which approach to use when beginning a project can be daunting. The most useful approach can be determined by asking the following questions: (1) What is the biological question? (2) What are the characteristics of the biological material? (3) What resources are available? Below we will discuss how each question influences the choice of technique.

### 4.1 What is the biological question?

The first question to be asked is quite simple, what kind of informational output is needed from the single‐cell experiment? As cancer progression involves complex dis‐regulation of multiple regulatory layers, many aspects of these mechanisms could be interrogated. Questions which seek to understand how tumor evolution alters the epigenome over time may benefit from a single‐cell method that uses one modality in the read‐out to simplify the resulting data analysis and interpretation (see Figure 2, green dots). If the question is more complex, for example how changes in chromatin accessibility or specific hPTM patterns influence gene expression during tumorigenesis, then multi‐omic or multi‐modal techniques are preferred (see Figure 2, blue dots). Obtaining multiple readouts per cell might not be desirable, as there is often a trade‐off between the number of modalities profiled and the coverage per modality (see Table 1—max reported reads per cell), as well as complications when interpreting the data due to the curse of dimensionality.^78^ However, some experimental systems are so complex that they might necessitate a multi‐omic readout to identify separate cell subpopulations.

### 4.2 What are the characteristics of the biological material?

Regardless of the biological question, the choice of method may also be influenced by the sample material itself. The amount of starting material available is often the most difficult and restrictive parameter for choosing a technique, as starting cell number requirements for single‐cell methods vary considerably by technique. Some reported cell requirements include: 150,000 cells for scCut&Tag^37^; 200,000 cells for sortChIC^55^; and 10,000 cells for CoBATCH.^39^ Minimum cell requirements are thus quite restrictive for rare samples like metastases or small biopsies, limiting the options for single‐cell studies. However, overcoming the cell minimum can be achieved with some alterations. For example, if the cell number is achievable through multiplexing of multiple experimental replicates or samples, a wider range of methods are usable. Multiplexing can be performed through the incorporation of unique cellular dyes^43^, ^45^ for each sample, combined into one tube for upstream experiment steps, and then index sorted using fluorescence‐activated cell sorting (FACS) into 384 well plates. However, this option is only viable with plate‐based approaches. Other options to overcome cell number restrictions can include the use of carrier cells to hit minimal numbers, but will eventually require removal so as not to compete with the sample of interest for sequencing.

Some single‐cell techniques are ideal for low cell numbers but require genetic modification. For example, the antibody‐free DamID‐based methods were extensively utilized for low‐input samples in early mouse preimplantation development.^79^, ^80^ Despite the low cell numbers, the data output is high quality and does not suffer from as much sparseness as other techniques. However, the DamID construct must be expressed in the cells of interest either through genetic manipulation or transduction methods, so it is more difficult to apply this method to primary tissues. As a result, this technique is more suitable for organoids, cell lines, or animal‐based models in cancer research.

Another particular characteristic of the sample to keep in mind is how many distinct cell types or states are expected, and how heterogeneous you expect your cell populations to be (even within one cell type). Tumor populations can be highly complex, particularly in the form of patient‐derived samples.^81^, ^82^, ^83^ Commonly there will be a mix of tumor cells, immune cells, endothelial cells, and possibly other cell types from the surrounding microenvironment. Therefore, high‐throughput methods that allow for profiling of a larger number of cells might be better suited, increasing the chances of capturing all the different cell subpopulations present. Despite some presence of clonal expansion, most primary tissue will benefit from having a transcriptomic readout regardless of biological question to allow for the separation of cell types.

How the samples are to be stored after collection is also critical to consider. Many of the discussed methods work best on fresh or cryopreserved single‐cell suspensions. In particular, methods with a transcriptomic readout suffer greatly from certain fixation methods due to RNA degradation. This is more easily overcome when using cell lines or organoid samples, as the researcher has more control over sample collection. For primary tissue, ideally the collection method should be adapted to be compatible with the chosen protocol. If this is not possible, optimization with non‐precious samples is required. Recent techniques have performed scRNA‐seq on formalin‐fixed paraffin‐embedded tissue (FFPE),^84^ and adapted CUTAC in bulk (FFPE‐CUTAC).^85^ These advances are a promising start, and may soon allow for compatibility of FFPE with more methods discussed in this review.

### 4.3 What resources are available?

Finally, the capability to ultimately execute the experiments depends on what equipment and sequencing resources are accessible to the lab (see Table 1—specialized equipment requirements). Many of the techniques discussed rely on liquid handling robots and while technically possible, would not be feasible to pipette by hand. For example, techniques considered plate based usually require the following steps: single‐cell sorting via FACS into 96 or 384‐well plates; nanoliter dispension via liquid handling robots of reagents into wells; multiple 384‐well equipped PCR machines; pooling reservoirs; and cooling centrifuges. In contrast, methods using combinatorial barcoding alleviate the need for specialized equipment, but may still require use of FACS for upstream processing depending on your starting material. Several commercial platforms exist such as 10X genomics (droplet‐based) or the ICELL8^86^ (nanowell dispensing) which are popular and easier to implement but require purchase of expensive kits or instruments.

The availability of sequencing machines should also be considered. Many institutions have in‐house sequencing cores with commonly used Illumina sequencers such as NextSeq, NovaSeq, etc. If no sequencing core exists in the institution, an outside company will be necessary with the caveat of longer return times and possibly higher costs. Discussions with the chosen sequencing option should include ideal sequencing depth for your sample type, compatible library preparations/barcodes, pooling parameters, length of the read, and if paired or single end sequencing is required. Ideally these conversations should occur before beginning an experiment as incompatibility of library preparation and sequencer can stall the experiment or even require repeated preparations and thus wasted materials and costs. Finally, commonly used equipment for quality control of library preparations pre‐sequencing is absolutely necessary. Quality control steps include precise measurement of library concentration (with Qubit 4 fluorometer for example), and a sensitive electrophoresis system (such as the Agilent TapeStation Systems) to determine library size and molarity of each sample. Once the sequencing libraries are completed, raw output files will require further processing. This will be discussed more below.

## 5 COMPUTATIONAL ANALYSIS AND CONSIDERATIONS

The analysis of single‐cell data is complicated due to the problem of data sparsity and the existence of background noise. These problems are accentuated in the field of single‐cell epigenomics due to the increase of both dropout events and biologically relevant zeroes, as well as the existence of method‐intrinsic biases. In the future, data sparsity may be bypassed computationally through imputation algorithms, as done for scRNA^87^, ^88^ and scATAC^89^, ^90^ data. Alternatively, in the case of multi‐omic data, the joint analysis of multiple molecular layers may allow for imputation or translation between modalities,^91^ or offer insights impossible to appreciate when studying exclusively one sparse epigenomic profile. Altogether, this makes the analysis of hPTM data particularly complex and highlights the need for dedicated personnel for the task. Additionally, the pre‐processing of single‐cell data can demand extensive computational resources, requiring access to high‐performance computing (HPC).

While the topic of analyzing single‐cell chromatin accessibility data has already been extensively covered,^92^, ^93^ there is a lack of consensus regarding computational approaches for single‐cell hPTM data, despite recent benchmarking efforts.^94^ Here we will provide a brief overview of the steps needed for the basic analysis of single‐cell hPTM data, covering data pre‐processing, downstream analyses, and integration with other datasets.

### 5.1 Data pre‐processing and downstream analyses

Before proceeding with the analysis of the epigenetic characteristics of each cell, certain pre‐processing steps are required to transform raw sequencing data into read count matrices. Firstly, the raw sequencing reads are allocated to different cells according to the barcodes introduced during the single‐cell tagging stage. These reads are mapped to a reference genome, counted, and aggregated into genomic bins of the appropriate size for the target and sequencing depth. Low‐quality cells and doublets are filtered according to parameters such as coverage or signal‐to‐noise metrics (like the proportion of non‐zero bins^55^ or information content^52^).

When working with data from different experimental batches, some of the variability might be caused exclusively by technical variation, a phenomenon known as “batch effects.” Sample multiplexing through approaches like hash barcoding^76^ not only help to reduce experimental costs, but also help to reduce batch effects caused by different reagent lots or sequencing runs. However, there might still be effects related to other factors like sample collection or storage. Depending on the technique used, different normalization approaches based on control datasets (open chromatin readouts or occurrence of genomic motifs) may be necessary before continuing with downstream analyses. Further technical variation may be removed computationally through batch‐correction, which will be covered further below.

Dimensionality reduction is used to transform a dataset to a lower dimensionality space while retaining as much relevant information as possible. It is an essential step in single‐cell data analysis, as it helps to reduce the noise and simplify the analysis, as well as making it easier to visualize and interpret complex data. While PCA is commonly used for dimensionality reduction in scRNA data,^93^ variations of both latent semantic indexing^56^ and latent Dirichlet allocation^55^ are also utilized for single‐cell hPTM data as alternative dimensionality reduction methods. The dimensionality‐reduced matrix can be used as input for visualization via UMAP or tSNA, or for clustering via algorithms such as Louvain or Leiden, in the same way as done for scRNA data.^93^

Once cell subpopulations are identified via clustering or other approaches, the readouts from the cells within the same subpopulation are aggregated into so‐called pseudo‐bulks.^95^ These are used for downstream analyses such as plotting patterns across genomic regions, peak calling (via either general peak‐calling algorithms like MACS3,^96^ or algorithms developed specifically for CUT&Tag data like GoPeaks^97^ or SEACR^98^), and visualization of enrichment over certain genomic regions. More complex analyses can be done using already‐published algorithms designed specifically for epigenomic data, like chromatin velocity,^99^ which allows the user to identify trajectories of epigenetic modifications at single‐cell level.

### 5.2 Data Integration

One of the great challenges of single‐cell data analysis is the integration of different datasets. Here we will briefly describe different types of data integration and their usage, but more in‐depth reviews that explore the mathematical and computational principles behind data integration have already been published.^100^, ^101^

One of the main concerns when integrating datasets covering the same modality from different cells are batch effects. Batch correction is used to remove this non‐biological variability by utilizing shared genomic features as “anchors” or “bridges” for integration. Many algorithms exist to perform batch correction in single‐cell data, like Seurat's MNN,^102^ Harmony,^103^ and scVI.^104^

It is also possible to integrate different modalities within the same set of cells to study the relationship and interactions between the different molecular layers. Algorithms like MOFA+^105^ enable the systematic study of associations between different hPTM patterns, or the relationship between changes in hPTM patterns and gene expression.

Both of these integration approaches require the existence of a shared “anchor” or “bridge” across datasets (either shared cell identity or shared genomic features across all cells). However, when dealing with complex tissues it might be useful to have more complex datasets, each generated from a different set of cells and containing different combinations of modalities. Mosaic data integration can be used to find a shared low‐dimensional space across all these datasets. While the field of single‐cell mosaic integration is still in its infancy, recent developments include StabMap^106^ and MIDAS.^91^

## 6 CONCLUSIONS

**FIGURE 3:**
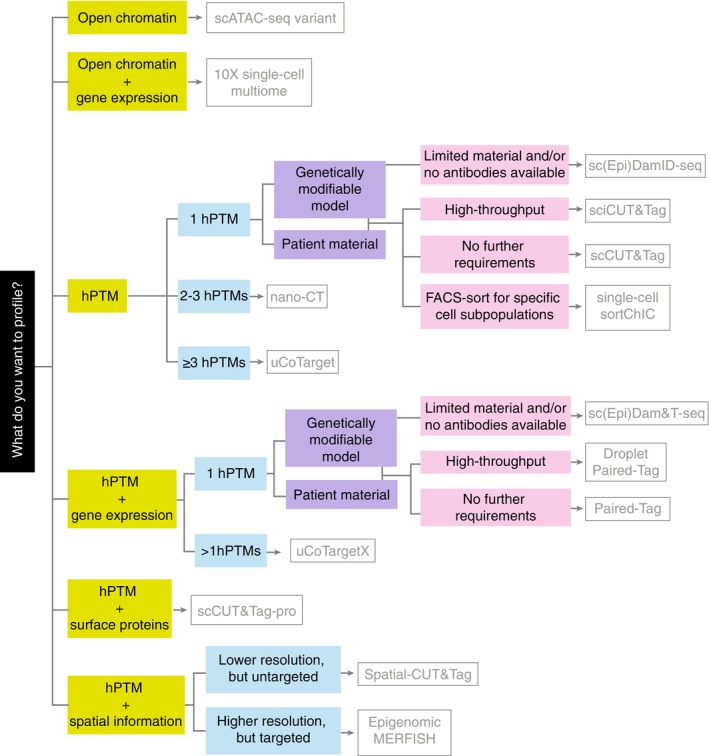
Decision flowchart for single‐cell method selection. Flowchart for choosing a method based on discussed considerations. As multiple methods might be suitable for a specific scenario, provided recommendations are based on the most popular methods currently used. However, other methods discussed in the text should still be considered and may prove more appropriate in specific contexts.

Applying single‐cell techniques to interrogate cancer biology is a necessary step forward for the field. These techniques provide insights into tumor heterogeneity unachievable through other methods, allowing identification of rare cell subpopulations and understanding of interactions between different molecular mechanisms within the same cell. However, the plethora of available single‐cell techniques can cause uncertainty and lead to a high barrier to entry. Here, we discussed various techniques (Figure 2) to interrogate epigenetic questions in the context of cancer biology. We further describe important metrics and requirements for each method in Table 1. To assist in choosing the best single‐cell method to pursue, we summarize parameters to consider in a flow chart (Figure 3). While the field is still developing, the wide range of techniques for single‐cell epigenomic profiling developed recently have the potential to provide groundbreaking knowledge of the role of epigenetic modifications in cancer.

Many of the discussed techniques have yet to be applied in a cancer context despite the important insights they could provide. For example, scCut&Tag was used to study H3K27me3 in brain tumor patients before and after treatment, revealing both heterogeneity within tumors as well as changes in the activity of polycomb complexes before and after treatment.^107^ Epigenetic modifiers might therefore be interesting both as predictors for treatment response as well as potential treatment targets. However, interpretation of response to treatment and selection of a target may be difficult with tumors containing significant heterogeneity. Thus, incorporating single‐cell multi‐omic techniques into both research and clinical approaches might provide key insights into cancer evolution. Multi‐omic techniques could even provide a more holistic profile using the same amount of material and directly tie changes to the epigenome with alterations in gene expression.

However, there are still several challenges in the field of single‐cell epigenomics that need to be addressed. Several aspects can be experimentally improved: (1) Most techniques depend on the existence of good‐quality antibodies, which might limit which marks can be studied; (2) there is a need for further development of low‐input approaches, as most techniques are not applicable in scenarios with limited material; and (3) specifically in the context of multi‐omic approaches, there is a trade‐off between the number of modalities and the coverage depth per molecular layer, which severely limits the sensitivity of these methods. Computationally, the field is still in its infancy, with a need for (1) standardization of computational pipelines for simple analyses, which has already been started by recent benchmarking approaches^94^; (2) the development of algorithms that can deal with the inherent sparsity and high dimensionality of single‐cell hPTM data, which depending on the binsize chosen can be much higher than that of scRNA or scATAC data, and (3) the development of algorithms that can handle the high complexity of single‐cell multi‐omic data and study the relationships between the multiple molecular layers.

## AUTHOR CONTRIBUTIONS

**Marta Moreno‐Gonzalez:** Conceptualization; writing – review and editing; writing – original draft. **Isabel Sierra:** Conceptualization; writing – review and editing; writing – original draft. **Jop Kind:** Supervision; writing – review and editing; funding acquisition.

## CONFLICT OF INTEREST STATEMENT

The authors declare the existence of a competing interest. Jop Kind is one of the inventors on a patent application (PCT/NL2022/050635, applicant: Koninklijke Nederlandse Akademie Van Wetenschappen) related to the MAbID technology and a co‐founder of sCellgen. The other authors declare no competing interests.
